# Risk assessment of farmers handling pelleted seeds containing crystalline silica and attapulgite

**DOI:** 10.1002/1348-9585.12304

**Published:** 2021-12-10

**Authors:** Mitsugu Hirano, Etongola Papy Mbelambela, Masamitsu Eitoku, Naw Awn J‐P, Yukiko Iida, Masaichi Terada, Narufumi Suganuma

**Affiliations:** ^1^ Department of Environmental Medicine Kochi Medical School Kochi University Nankoku Kochi Japan; ^2^ Environmental Control Center Co. Ltd Hachioji, Tokyo Japan; ^3^ Japan Seed Trade Association Bunkyo, Tokyo Japan

**Keywords:** attapulgite, farmers, pelleted seeds, personal exposure measurements, risk assessment, silica

## Abstract

**Objectives:**

This study aimed to assess the risk to farmers from handling pelleted seeds that include crystalline silica and attapulgite.

**Methods:**

We measured personal exposure levels to respirable crystalline silica and attapulgite in the experimenter representing a farmer in a simulated workplace. From these values, the annual occupational exposure levels were estimated and compared with the established occupational exposure limits. To assess the toxicity of respirable crystalline silica and attapulgite, digital chest images of workers in a factory producing pelleted seeds were examined.

**Results:**

The personal exposure measurement results showed that the concentrations of total dust, respirable dust, and respirable crystalline silica generated during work handling of pelleted seeds were 0.27, 0.06, and 0.00043 mg/m^3^, respectively. The estimated annual occupational exposure level to total dust, respirable dust, and respirable crystalline silica in farmers was 10^3^ to 10^4^ times lower than established occupational exposure limits. Attapulgite was not detected by analysis of the pelleted seeds themselves or dust collected during the personal exposure measurements. No pulmonary parenchymal or pleural lesions were detected in the digital chest images of the factory workers.

**Conclusion:**

We found that farmers handling pelleted seeds would not be exposed to levels of total dust, respirable dust, respirable crystalline silica, and attapulgite derived from pelleted seeds exceeding occupational exposure limits. These results suggest that the risk to farmers of handling pelleted seeds is negligible.

## 1 INTRODUCTION

In the agricultural workplace, exposure to mixtures of organic and inorganic dust is common. Organic dust originating from plant and animal sources commonly causes allergic diseases such as asthma. Inorganic dust originating predominantly from the soil tends to cause non‐allergic reactions in the lungs. Soil contains two chemicals associated with lung disease. Silica is associated with respiratory conditions in people in agricultural occupations,^1^, ^2^, ^3^ and the fibrous mineral attapulgite can cause fibrotic changes in human lungs.^4^

Seeds of various shapes and sizes are coated with clay minerals and processed into spherical shapes of uniform size suitable for efficient seeding by sowing machines. These pelleted seed products contain both crystalline silica and attapulgite. In Japan, an amendment to the Industrial Safety and Health Law in 2016 made risk assessment mandatory for 640 chemicals, including crystalline silica, and for products containing those chemicals. In addition, because the risk from attapulgite‐containing products has not been previously assessed by manufacturers, the risk due to the attapulgite contained in pelleted seeds also needs to be assessed.

In the present study, we aimed to investigate whether farmers handling pelleted seeds are exposed to respirable dust, respirable crystalline silica, and attapulgite and whether they are therefore at risk for developing lung parenchymal and interstitial lesions. The study results will provide benchmarks useful for future occupational risk assessments related to grain dust exposure in Japan and other countries where exposure conditions are similar.

## 2 METHODS

### 2.1 Study design

**FIGURE 1:**
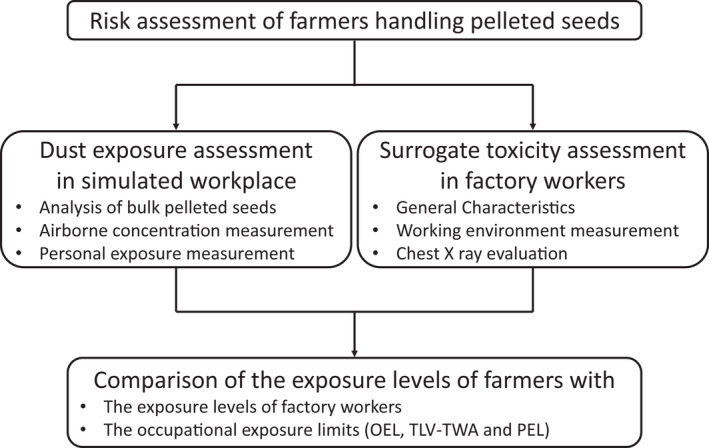
Schematic diagram representing study design for risk assessment for farmers handling pelleted seeds. Exposure to and toxicity from attapulgite, total dust, respirable dust, and respirable crystalline silica generated from pelleted seeds were assessed. To assess exposure levels to silica and attapulgite from pelleted seeds rather than from the soil, workplace and personal exposure levels were measured in a simulated workplace. For the toxicity assessment, workers in pelleted seed factories who had been exposed to specific dusts in large amounts over a long period of time were studied as surrogate of farmers. The exposure levels in farmers were then compared with those in factory workers and various established occupational exposure limits

**FIGURE 2:**
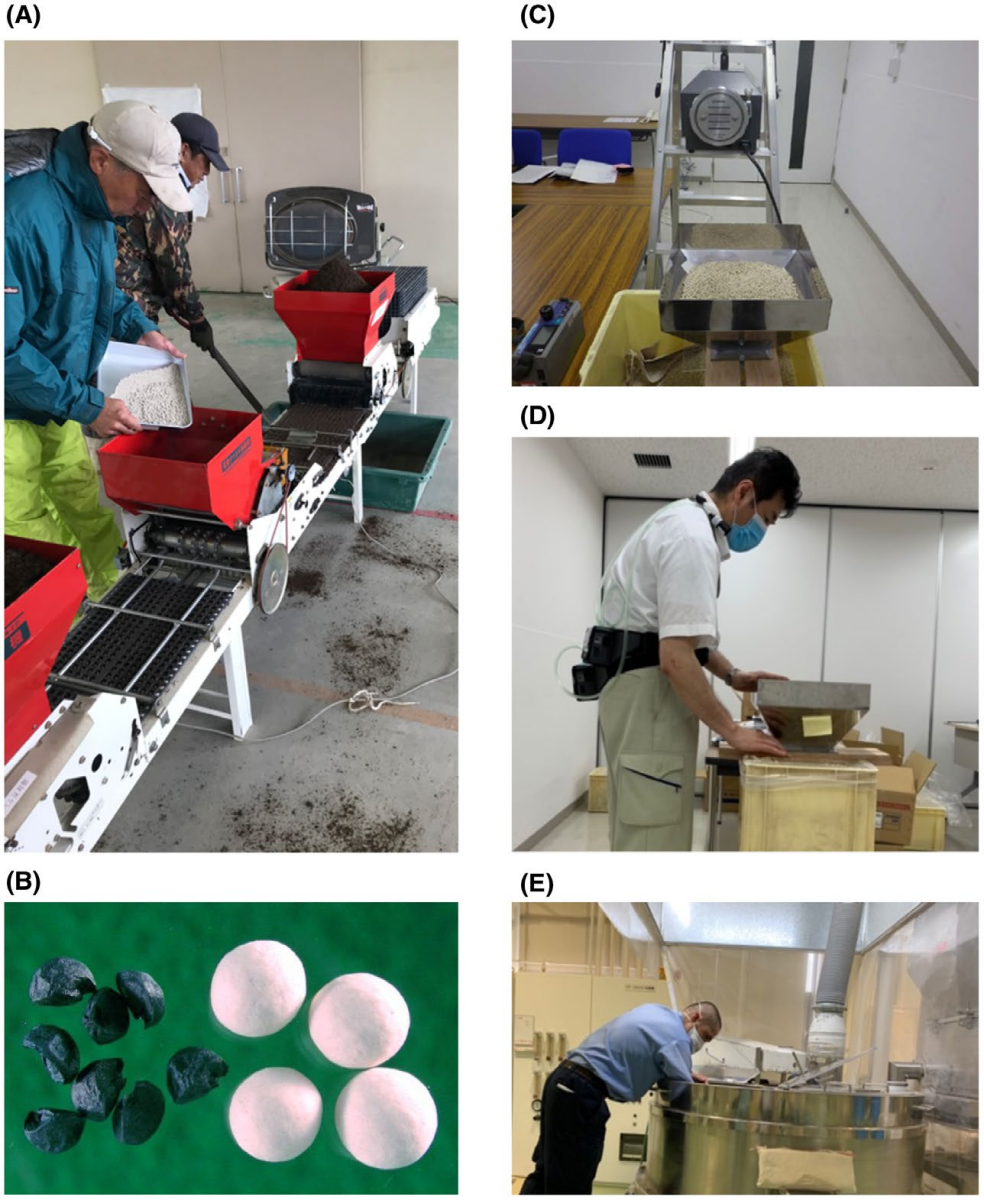
Experimental setting and observational subject for risk assessment. (A) Farmer handling pelleted seeds in the equipment garage, the subject of risk assessment in this study. (B) Appearance of pelleted seeds and original onion seeds. (C) Airborne concentration measurement with a high‐volume sampler. (D) Personal exposure measurement with personal samplers. (E) Factory workers working in the pelleting process of the seeds wearing disposable masks. Local air exhausters installed over the head is operated during the pelleting work

Because it is difficult to evaluate the risk posed by exposure to pelleted seeds themselves in farmers, who are also exposed to soils containing crystalline silica and attapulgite, the exposure assessment was conducted in a simulated workplace, and a separate toxicity assessment was conducted in workers in pelleted seed factories in Japan (Figure 1). Annual occupational exposure levels in farmers, estimated from the results of personal exposure measurements conducted in the simulated workplace, were then compared with annual occupational exposure levels in factory workers and annual exposure limits calculated using various established occupational exposure limits such as the occupational exposure limit for chemical substance (OEL) established by the Japan Society for Occupational Health (JSOH), the threshold limit value–time‐weighted average (TLV‐TWA) established by the American Conference of Governmental Industrial Hygienists (ACGIH), and the permissible exposure limit (PEL) established by the U.S. Occupational Safety and Health Administration (OSHA). Pelleted onion seeds were used in this study because onion farmers handle them in large amounts in closed workplaces (Figure 2A,B). Prior to the personal exposure measurements, airborne concentrations during handling pelleted seeds in the simulated workplace were measured to determine the experimental conditions (Figure S1). Toxicity was assessed in factory workers producing pelleted seeds as a surrogate of farmers. The exposure level of the factory workers to dust was estimated by using working environment measurement data provided by the factory. From these values, the annual occupational exposure levels of factory workers were estimated and compared with those of farmers. To assess the health of the factory workers, chest X‐rays of workers, obtained during their general health checkups, were evaluated for interstitial or pleural abnormalities.

### 2.2 Materials and subjects

There were altogether five palleted seeds production companies in Japan, denoted as A–E. We obtained pelleted seed samples from all the companies, analyzed the silica and attapulgite contents in the coating materials, and measured the airborne concentration in the simulated workplace. After comprehensive consideration of distribution amounts of the pelleted seeds, the amount of dust generated during their handling, and the silica content of the dust, we used pelleted seed samples from two companies (A and B) for the personal exposure measurements in the simulated workplace. At the time this study was conducted, one company stopped production. Therefore, 31 male workers who work at pelleted seed production departments from the remaining four companies were enrolled in the toxicity assessment.

### 2.3 Exposure assessment in the simulated workplace

Pelleted seeds samples were crushed to analyze their silica and attapulgite contents (Figure 2B). In the classification system of the International Agency for Research on Cancer (IARC), attapulgite is a Group 2B substance that is possibly carcinogenic to humans, but only if the fiber length is longer than 5 µm.^5^ The presence of attapulgite in the coating materials was determined following the standard method for asbestos analysis (JIS A1481‐2; 2016) by using dispersion staining‐phase contrast microscopy (BX‐51 microscope; Olympus Corporation) and X‐ray diffractometer (X'pert^3^ Powder; PANalytical) (Table S1).^6^ In the dispersion staining‐phase contrast microscopy analysis, the immersion oil with a refractive index of 1.550, which is the same as that for chrysotile, was used for attapulgite detection because the refractive index of the immersion oil in which the attapulgite shows a dispersion color is 1.500–1.560.^6^, ^7^ The silica content of the coating materials of pelleted seeds was analyzed by X‐ray diffraction with an X’pert PRO MPD system (PANalytical).

For the assumption on the side of safety, a small meeting room in a factory with an air volume of 67.2 m^3^ (room area 28.0 m^2^; ceiling height 2.4 m), where the temperature and humidity were set at 20–25°C and 40%–70% during measurements, was used to simulate a relatively larger garage where farmers handle pelleted seeds. The pelleted seed samples from the five factories were tested independently. In each test, a total of 330 000 pelleted seeds were placed in a pseudo‐device simulating a standard seeding machine (High Speed Onion Spreader OSE‐110; Minoru Industrial Co. Ltd) (Figure 2C, Figure S1A,B) for 1 h under intermittent operation in multiple batches. During each test, respirable dust was collected and measured at a height of 1200 mm and at a distance of 300 mm from the dust source, with the operator in a standing position (Figure S1C). To avoid cross‐contamination errors, after each test, the next test was not begun until the respirable dust level was 0.001 mg/m^3^ or less, as measured by a digital dust indicator (LD‐3K2; Sibata Scientific Technology Co. Ltd). The dust generated by each sample was collected by a high‐volume air sampler (HVS; HV‐500F, Sibata Scientific Technology Co. Ltd) using an air sampling volume of 30 m^3^ in 1 h test (Table S1). A particle size separator (HV500PM4; Sibata Scientific Technology Co. Ltd) was attached to the HVS to separate respirable dust. The airborne concentration of respirable dust was calculated by dividing the weight of the collected dust, measured by a gravimetric electronic scale (HM202; A&D Co. Ltd), by the sampled air volume. The silica content of the respirable dust was analyzed by using the X’pert PRO MPD (PANalytical).

Personal exposure to attapulgite, total dust, and respirable dust was measured in the same experimental setting as the airborne concentration measurement by using personal samplers (Figure 2D and Table S1). The experimenter representing a farmer placed the pump at waist level and the sampler in the neck area, near where the air is breathed in (Figure S1D). A personal sampler (MP‐W5P; Sibata Scientific Technology Co. Ltd) was used for the sampling of attapulgite, total dust, and respirable dust. Attapulgite was collected using a disposable asbestos sampling filter holder (225–321; SKC Ltd) with a membrane filter with an effective diameter of 25 mm and pore size of 0.8 μm. Total dust, and respirable dust were collected using a filter holder with an effective diameter of 19–20 mm (NWPS‐254; Sibata Scientific Technology Co. Ltd) separating particles by inertial collision particle size selection (4 μm, 50% cut). The sampling flow rate was 1 L/min for attapulgite and 2.5 L/min for total dust, and respirable dust. The sampling time in each case was 8 h, which was estimated to be sufficient for dust sampling above the detection limit based on the previous airborne concentration measurement results obtained in the simulated workplace. Total dust was calculated by combining the weight differences, before and after sampling, of the impact plate and the filter (TF98R; Sibata Scientific Technology Co. Ltd). Before sampling, grease applied impact plate and filters were placed in an environment controlled by temperature and humidity and then weighed with the gravimetric electronic scale (MC‐5; Sartorius AG). After sampling, the impact plates and filters were placed for one day in the same environment before weighing. For measurement of respirable dust, the filter was weighed again one day after weighing. Since the respirable dust quantity was very minute and the effect of moisture was bigger than total dusts, the measured weight within measurement error ±0.003 mg from the previous day was taken as the weight of respirable dust. The personal exposure levels of total dust and respirable dust were calculated by dividing the weight of the collected dust by the sampled air volume. The presence of attapulgite in the air was determined following the standard method for asbestos analysis (JIS K 3850‐1; 2006) by using dispersion staining‐phase contrast microscope (BX‐51 microscope; Olympus Corporation).^7^

### 2.4 Estimation of the annual occupational exposure level of farmers and annual exposure limits

The annual exposure levels of farmers to attapulgite, total dust, and respirable dust were estimated separately by multiplying the personal exposure concentration of the experimenters representing farmers by the estimated respiratory volume and the estimated annual number of working hours of farmers. The respirable crystalline silica concentration was calculated by multiplying the respirable dust concentration by the silica content of the respirable dust sampled in the airborne concentration measurement.

Applying the tidal volume of 1.25 L and breathing frequency of 20 cycles/min occurred in light‐work breathing,^8^ the respiratory volume of a farmer was calculated: 1.25 L × 20 cycles/min × 60 min = 1500 L (=1.5 m^3^).

The annual number of working hours of farmers was estimated as follows. The estimated cultivated area of 10 ha per onion farmer requires about 3.2 million pelleted seeds.^9^, ^10^ Because the amount of seeding in one hour by the high speed seeding machine was 330 000, which is almost equal to the number of seeds required for 1 ha, the annual number of hours that farmers work with pelleted seeds was estimated to be about 10 h.

Annual exposure limits were calculated by multiplying the established limits (OEL, TLV‐TWA and PEL) by annual respiratory volume. Respiratory volume was set as 1.5 m^3^, then the annual respiratory volume can be calculated as:

$$1.5{\;m}^{3}/h\times 8\;h/\text{day}\times 5\;\text{days}/\text{week}\times 48\;\text{weeks}=2880{\;m}^{3}$$

### 2.5 Working environment measurement in pelleted seed production departments of factories

Although the worldwide standard method used to ensure the protection of workers from exposure to chemicals requires personal exposure assessment in the workplace, in Japan, to protect the health of workers and promote environmental improvement, work environment measurements are carried out.^11^ To evaluate the concentration of floating dust in the air in the work environment, two types of measurement, Type A and Type B sampling, were carried out. Type A sampling, which was used to assess average spatial and temporal variations in the airborne dust concentration in the workplace, provides two evaluation concentrations called 1st and 2nd evaluation values, i.e., the 95th percentile concentration and the arithmetic mean concentration, respectively.^12^, ^13^ Type B sampling was used to assess the concentration at the place and time when the exposure of workers was considered to be maximum, because Type A sampling may miss exposures of a worker to high concentrations due to, for example, the emission of a toxic substance or an individual's work posture or methodology.

The pelleted seed production process comprises seed selection, pelleting, drying, sizing, checking, and packing steps. Pelleting is a wet operation during which moist powdered materials are sprayed onto the seeds. Afterward, the pellets must be dried to complete the pelleting process.^14^ In the case of onion seeds, the dried, pelleted seeds are put into a sorting apparatus to select pelleted seeds in a diameter range of 3.5–4.5 mm. During the pelleting and sizing processes, which generate dust, local air exhausters are installed and operated (Figure 2E). The airborne concentration of the "floating dust" in the work environments where these processes take place is measured twice a year following Japanese protocols of working environment measurement.^12^, ^13^

### 2.6 Estimation of annual occupational exposure levels of workers producing pelleted seeds

When the value of the working environment measurement is used as a substitute value for the personal exposure concentration, it is reasonable to use the Type B sampling value, which is higher on the safe side.^11^, ^15^ However, in this paper, the hazard is estimated together with the results of workers’ health checkups. If we use the Type B sampling value which may be a higher concentration than the actual exposure concentration, as a result, the health hazard will be underestimated. Therefore, in this paper, the 2nd evaluation value of the Type A sampling (arithmetic mean value) is used as the estimated value of 8‐h TWA.^15^, ^16^ The annual exposure of workers to respirable dust was estimated by multiplying the 2nd evaluation values by the annual number of working hours of workers producing pelleted seeds in a factory. We considered "floating dust" measured in the work environment to be synonymous with "respirable dust".

### 2.7 Toxicity assessment in pelleted seed factories

The toxicity of pelleted seeds to workers in pelleted seed factories was evaluated. Four companies provided information on 31 workers, including their age, sex, job tenure, and digital chest images. All of these workers had previously worked or currently worked in the pelleted seed production department of the companies and had had chest X‐rays taken between 2017 and 2019 during their annual health checkups. At present, workers in pelleted seed production departments of these factories work 2–6 h per day, 4–5 days a week. While they work, they wear disposable masks specifically designed for protection against dust during dust‐producing processes. The most recent digital chest images of each worker were independently reviewed by two occupational health physicians: a U.S. National Institute for Occupational Safety and Health (NIOSH)‐certified B Reader with over 20 years of experience and an AIR Pneumo‐certified reader with over 5 years of experience.^17^ The presence of dust‐related interstitial or pleural abnormalities was recorded using the International Labor Office Classification of Radiographs of Pneumoconiosis system.^18^

## 3 RESULTS

The silica contents of bulk pelleted seeds ranged from 4.3% to 17.2%, and no attapulgite was detected (Table 1). The respirable dust concentration of the air ranged from 0.02 to 0.06 mg/m^3^ (Table 1). The silica content of the respirable dust sampled during the work handling of the pelleted seeds was <3%, and it was classified into Class 2 dust according to the JSOH classification.^19^ The OELs for Class 2 dust are 4 mg/m^3^ (total dust) and 1 mg/m^3^ (respirable dust).

**TABLE 1:** Exposure assessment results and corresponding exposure limits for attapulgite, total dust, respirable dust, and respirable crystalline silica for experimenters representing farmers handling pelleted seeds in the simulated workplace

	A	B	C	D	E	OEL (JSOH)	TLV‐TWA (ACGIH)	PEL (OSHA)
Analysis of bulk pelleted seeds								
Attapulgite	Not detected	Not detected	Not detected	Not detected	Not detected	—	—	—
Silica content (%)	5.0	4.3	6.0	4.3	17.2	—	—	—
Airborne concentrations measured in the simulated workplace								
Respirable dust (mg/m^3^)	0.06	0.03	0.03	0.03	0.02	—	—	—
Silica content (%)^a^	0.72	<0.56	<0.51	<0.56	<0.28	—	—	—
Respirable crystalline silica (mg/m^3^)	0.00044	<0.00014	<0.00014	<0.00015	<0.00005	—	—	—
Personal exposure measured in the simulated workplace								
Attapulgite (fibers/L)^b^	<0.5	<0.5	No data	No data	No data	150^c^ (Chrysotile)	100 (Chrysotile)	100 (Chrysotile)
Total dust (mg/m^3^)	0.27	0.06	No data	No data	No data	4 (Class 2)	—	30/(Q+2)
Respirable dust (mg/m^3^)	0.06	0.05	No data	No data	No data	1 (Class 2)	3	10/(Q+2)
Silica content (%)	0.72	<0.56	No data	No data	No data	—	—	—
Respirable crystalline silica (mg/m^3^)	0.00043	<0.00028	No data	No data	No data	0.03	0.025	—

Abbreviations: ACGIH, American Conference of Governmental Industrial Hygienists; A–E, companies providing pelleted seeds; Class 2, dust containing <3% crystalline silica; JSOH, Japan Society for Occupational Health; OEL, occupational exposure limit for a chemical substance; OSHA, Occupational Safety and Health Administration; PEL, permissible exposure limit; Q, crystalline silica content (%); TLV‐TWA, threshold limit value–time‐weighted average.

^a^ The measured silica content of pelleted seeds from companies B–E was less than the detection limit.

^b^ Detection limit was <0.5 fibers/L.

^c^ The reference value of chrysotile recommended by the JSOH was defined as the excess lifetime risk of cancer attributable to exposure to chrysotile during 40 years of work.^19^

Personal exposure levels were measured using samples from companies A and B for two reasons. First, the sample provided by company A showed the relatively highest respirable dust concentration and silica content. Second, of the remaining samples showing similar respirable dust concentrations and silica content below the detection limit, the sample provided by company B had a high market share of pelleted seeds. As shown in Table 1, the highest concentrations of total dust, respirable dust, and respirable crystalline silica generated during the work handling of pelleted seeds were 0.27, 0.06, and 0.00043 mg/m^3^, respectively. These concentrations thus did not exceed their respective OELs.

**FIGURE 3:**
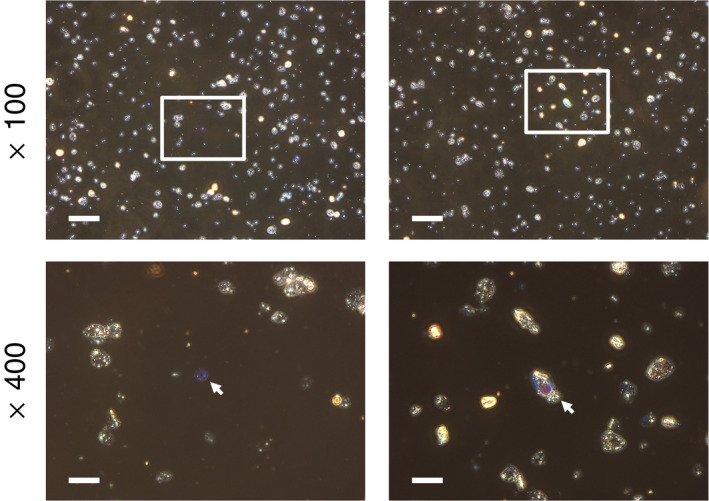
Morphological features of attapulgite particles collected on the filters used in the personal exposure measurement. Samples were examined using an immersion oil with a refractive index of 1.550. The images were observed using dispersion staining‐phase contrast microscopy with ×100 (upper) and ×400 (lower) lenses. Left and right images show the samples provided by company A and B, respectively. White bars in the upper and lower images indicate 80 and 20 μm, respectively. The rectangles in the upper images are the visual fields of the lower images. The white arrows in the lower images indicate examples of particles showing the dispersion color of blue

As shown in Figure 3, dispersion staining‐phase contrast microscopy indicated that a few particles showed the dispersion color of red‐purple or blue (refractive index = 1.550), but no fibrous particles showing the dispersion color were detected in both samples.

The work environment measurement results used to estimate the exposure levels of factory workers to respirable dust and respirable crystalline silica are summarized in Table 2.

**TABLE 2:** Toxicity assessment of workers in pelleted seed factories

	Factory workers (*n* = 31)
Respirable dust concentration in workplace	
1st evaluation value (mg/m^3^): median (min–max)	0.26 (0.02–1.11)
2nd evaluation value (mg/m^3^): median (min–max)	0.10 (0.01–0.46)
B‐sampling value (mg/m^3^): median (min–max)	0.14 (0.01–0.97)
Respirable crystalline silica concentration in workplace	
1st evaluation value × silica content (mg/m^3^): median (min–max)	0.0028 (0–0.0151)
2nd evaluation value × silica content (mg/m^3^): median (min–max)	0.0011 (0–0.0056)
Type B sampling value × silica content (mg/m^3^): median (min–max)	0.0009 (0–0.0092)
Age (years): mean (SD)	37.7 (7.9)
Male sex, *n* (%)	31 (100%)
Job tenure (years): mean (SD)	8.6 (6.7)
Pulmonary parenchymal lesions: *n* (%)	0 (0%)
Pleural lesions: *n* (%)	0 (0%)

Note

1st evaluation value, estimated value that is the 95th percentile concentration of airborne toxic substances during working hours at all possible points in the work area; 2nd evaluation value, estimated arithmetic mean concentration of airborne toxic substances in the work area; Type B sampling value, concentration at the place and time when the exposure of workers was considered to be a maximum.^12^, ^13^

The estimated annual occupational exposure levels of farmers were lower than both those of the factory workers and the annual exposure limits calculated from the various established occupational exposure limits. The highest estimated annual occupational exposure levels of farmers for products of the two companies were 4.1 × 10^0^, 9.0 × 10^–1^, and 6.5 × 10^–3^ mg/year for total dust, respirable dust, and respirable crystalline silica, respectively. The estimated annual occupational exposure levels of factory workers calculated using 2nd evaluation values ranged from 4.0 × 10^0^ to 6.6 × 10^2^ mg/year for respirable dust and from 0.0 to 8.1 × 10^0^ mg/year for respirable crystalline silica. Among the annual exposure limits calculated using OEL (JSOH), TLV‐TWA (ACGIHP), and PEL (OSHA) values, the lowest indicated limits for total dust, respirable dust, and respirable crystalline silica were 1.2 × 10^4^, 2.9 × 10^3^, and 7.2 × 10^1^ mg/year, respectively (Table 3).

**TABLE 3:** Comparison of estimated annual occupational exposure levels with occupational exposure limits

	Estimated annual occupational exposure level			Estimated annual occupational exposure limit		
	Farmers^a^		Factory workers^b^	OEL^c^	TLV‐TWA^c^	PEL^c^
	Pelleted Seed (Factory A)	Pelleted Seed (Factory B)				
Attapulgite (fibers/year)	<7.5 × 10^3^	<7.5 × 10^3^	No data	4.3 × 10^8^ d (Chrysotile)	2.9 × 10^8^ (Chrysotile)	2.9 × 10^8^ (Chrysotile)
Total dust (mg/year)	4.1 × 10^0^	9.0 × 10^–1^	No data	1.2 × 10^4^	—	3.2 × 10^4^
Respirable dust (mg/year)	9.0 × 10^–1^	7.5 × 10^–1^	3.8 × 10^0^–6.6 × 10^2^	2.9 × 10^3^	8.6 × 10^3^	1.1 × 10^4^
Respirable crystalline silica (mg/year)	6.5 × 10^–3^	<4.2 × 10^–3^	0.0–8.1 × 10^0^	8.6 × 10^1^	7.2 × 10^1^	—

Abbreviations: OEL, occupational exposure limit for a chemical substance; PEL, permissible exposure limit; TLV‐TWA, threshold limit value–time‐weighted average.

^a^ Estimated annual occupational exposure level of farmers: personal exposure concentration × respiratory volume (1.5 m^3^/h) × estimated annual working hours of onion farmers (10 h/year).

^b^ Estimated annual occupational exposure levels of factory workers calculated using the 2nd evaluation values: minimum value × 2 h/day × 4 days/week × 48 weeks; maximum value × 6 h/day × 5 days/week × 48 weeks.

^c^ Annual occupational exposure limits estimated as follows: occupational exposure limit × respiratory volume (1.5 m^3^/h × 8 h/day × 5 days/week × 48 weeks).

^d^ Estimated using the reference value of chrysotile recommended by the JSOH, defined as the excess lifetime risk of cancer attributable to exposure to chrysotile during the normal 40 years of work.

Among the personal exposure levels to attapulgite, total dust, respirable dust, and respirable crystalline silica, only that of attapulgite was below the detection limit. Annual exposure of farmers to attapulgite was therefore estimated using the lower limit of quantification of the personal exposure measurements. Because the detection limit was 0.5 fibers/L, the actual concentration could be as high as 0.48 fibers/L or as low as 0 fibers/L, but 0.5 fibers/L was used to calculate the annual exposure for the safety assessment. The estimated annual occupational exposure level of farmers to attapulgite was <7.5 × 10^3^ fibers/year (Table 3). Because there is no OEL for attapulgite, this estimated value was compared to the reference value of chrysotile, which is classified as a Group 1 substance (carcinogenic to humans) by the IARC and has the same fibrous nature as attapulgite. The reference value of chrysotile set by the JSOH, which is defined as the excess lifetime risk of cancer attributable to exposure to chrysotile during 40 years of work, is 150 fibers/L.^19^ This means that exposure to 150 fibers/L of chrysotile during 40 years of work would result in one excess cancer case per 1000 workers.^20^ The annual exposure limit estimated by using the JSOH definition for the carcinogenicity of chrysotile was 4.3 × 10^8^ fibers/year, whereas the limits estimated by using TLV‐TWA and PEL values for chrysotile were both 2.9 × 10^8^ fibers/year.

The toxicity assessment results showed that the mean age of the 31 workers was 37.7 years, and their mean job tenure was 8.6 years (Table 2). All of the workers were male, and no parenchymal or pleural lesions were detected on their digital chest X‐ray images.

## 4 DISCUSSION

To our knowledge, this is the first study to show that the risk to farmers handling pelleted seeds containing crystalline silica and attapulgite evaluated by personal exposure measurement is negligibly small. The estimated annual exposure levels of farmers were first compared with those of factory workers and then with the annual occupational exposure limits derived from various exposure limit recommendations. We estimated the annual exposure levels of respirable crystalline silica to be 6.5 × 10^–3^ and <4.2 × 10^–3^ mg/year in farmers, whereas the level was 0.0–8.1 × 10^0^ mg/year in factory workers (Table 3). Therefore, the annual exposure levels of farmers are approximately 1000–2000 times lower than that of factory workers. No factory workers revealed parenchymal or pleural abnormalities, suggesting the risk of adverse respiratory health was lower in farmers handling pelleted seeds. The annual exposure to respirable crystalline silica estimated from the measurement results was 10^4^ times lower than the annual exposure level estimated using OEL (JSOH) and TLV‐TWA (ACGIH) values, suggesting that the health risk to farmers of respirable crystalline silica derived from pelleted seeds is very small (Table 3). We noted a discrepancy of silica contents in the bulk and respirable dust samples between pelleted seeds provided by A and E companies. A possible explanation might be non‐inhalable crystalline silica with an aerodynamic particle size of 4 μm or larger does not fly in the air for a long time and falls quickly by gravity.

The lifetime excess cancer risk from attapulgite fiber derived from pelleted seeds was also negligible. In our analysis results, no attapulgite with a fiber length exceeding 5 μm ("possibly carcinogenic" according to the IARC) was detected in the pelleted seeds. Because fine fibrous materials such as attapulgite tend to disperse in the atmosphere, when a large amount of pellets are handled, dispersed attapulgite may be present even though no attapulgite was detected by the analysis of the pellet itself. Therefore, we measured the personal exposure concentration for 8 h during the simulated sowing of pelleted seeds. However, no attapulgite was detected in these measurements. It is possible that attapulgite contained in the raw material lost its fibrous structure as a result of heating and stirring during the manufacturing process. Because the possibility that attapulgite was present at concentrations below the detection limit could not be ruled out, we estimated the annual exposure level to attapulgite by comparing the detection limit for the personal exposure measurement with the estimated allowable exposure to chrysotile (asbestos), which is a fibrous mineral with higher carcinogenicity than attapulgite. The resulting estimated excess lifetime cancer risk due to exposure to attapulgite was 0.002 cases per 10^5^ persons. This lifetime excess cancer risk is well below the target of one case per 10^5^ workers established by Japanese Ministry of the Environment.^21^ Therefore, the health risk to farmers from attapulgite derived from pelleted seeds can be considered to be very small.

To assess the risk to farmers handling pelleted seeds containing crystalline silica and attapulgite, we believe that measurement of personal exposure is the most appropriate assessment method. There are several risk assessment methods, but the accuracy of the evaluation differs depending on the method. Control banding is one of the risk assessment methods that does not need the expert's exposure measurement, but it has the disadvantage of estimating results of shifting to a safer side rather than actual risk.^22^, ^23^, ^24^ For this reason, in evaluations using control banding, many substances are subject to restrictions on the amount and time used or consideration of alternative substances.^22^ In the case of biological monitoring, it is not possible to assess the exposure level of hazardous substances if hazardous substances, their metabolites or related adducts in human urine or blood cannot be qualified or quantified. Moreover, specialized knowledge is required to obtain and analyze the requisite biological samples. In the case of crystalline silica and attapulgite, the metabolites that should be evaluated by biological monitoring have not been identified. In risk assessments using personal exposure measurements, the assessed risk is based on the actual exposure of workers, so more accurate evaluation of the actual health risk is possible.

The toxicity assessment results for factory workers producing pelleted seeds strengthen the risk assessment results obtained by personal exposure measurements. The absence of parenchymal and interstitial lesions might be attributable to low dust levels in the factory workplace and the short job tenures of the factory workers (Table 2). At the factory, workers wore nationally certified disposable masks. The disposable masks used in the pelleted seed factories have an assigned protection factor of 10,^25^, ^26^ and mask‐wearing is expected to reduce exposure to respirable crystalline silica to one‐tenth or less of the airborne concentration. As a result, the annual exposure level of factory workers producing pelleted seeds to crystalline silica (0.0–8.1 × 10^0^ mg/year) was 100 times lower than the annual exposure limit based on the TLV‐TWA value. Silicoproteinosis is known to occur after exposure times ranging from a few weeks to <5 years when the annual exposure levels to respirable crystalline silica are very high, on the order of 1–10 mg/m^3^ (approximately 2.9 × 10^3^ mg/year based on our estimated annual respiratory volume), but these exposure levels are unlikely to occur in the studied population.^27^

This study has several strengths. First, risk assessment of farmers who handle pelleted seeds was based not only on the results of personal exposure measurement in a simulated work environment but also on the toxicity assessment results of factory workers producing pelleted seeds and work environment measurements. Factory workers producing pelleted seeds are expected to be exposed to higher concentrations of dust for longer time periods than farmers. Second, this study assessed specific pollutants, namely, attapulgite, respirable dust, and respirable crystalline silica. Third, toxicity assessment was conducted on almost all workers previously or currently working in the production department of pelleted seeds factories in Japan. These factories were among the world's top 10 factories. Fourth, the digital chest images were independently examined by two occupational health physicians with long experience.

Nevertheless, our study also has some limitations. First, the exposure levels of farmers were not directly assessed in the simulation study, although the estimated exposure levels to respirable crystalline silica and attapulgite derived from pelleted seeds were negligible. The applicability of the present results to farmers should be carefully considered. Since the working environment of farmers may not be a completely closed environment, but is subject to wind effects, which can alter local airborne concentrations. In addition, the temperature and humidity in the actual condition might differ from our experiment. For example, farmers usually sow onion seeds in March in Hokkaido and September in Hyogo and Saga where are. According to the Japan Meteorological Agency, the average temperature and humidity for the past 30 years in September in Hyogo and Saga prefectures are 23.5–24.5°C and 72%–78%. In the case of Hokkaido, the respective figures in March are −1.3°C and 70%, but the temperature inside the garage may vary because heating appliances are sometimes used. Second, some may argue the reliability of the measured personal exposure levels because each sample was measured only once. However, independent measurements of airborne concentrations and personal exposure levels of respirable dust were similar, which assured the reliability of our measurements. Third, the toxicity of crystalline silica, and attapulgite derived from pelleted seeds was not evaluated in farmers but in factory workers, because the latter are exposed only to dust derived from pelleted seeds. Although the number of factory workers evaluated was small, almost all workers in production departments of pelleted seed factories in Japan were evaluated, so the selection bias was likely limited. Fourth, the smoking status of the factory workers is unknown. However, no lesions attributable to lung disease caused by smoking were observed on the chest X‐ray images, possibly because the factory workers were young. Their mean age was 37.7 years; their mean job tenure was 8.6 years. In contrast, the mean age of farmers in Japan is 57.2 years^28^ and farmers who begin farming at age 20 might work for as long as about 37 years. Because lung disease progression is irreversible, and the disease progresses even after exposure ceases,^3^ a follow‐up study of older workers who have worked longer than 8 years might yield different results. Fifth, since the OEL set by JSOH was aiming at preventing pneumoconiosis progression, not the occurrence, the annual exposure limit derived was not the best choice as a reference.

In conclusion, in the present study, we found that farmers handling pelleted seeds would not be exposed to levels of total dust, respirable dust, respirable crystalline silica, and attapulgite derived from pelleted seeds exceeding established limits (OEL, TLV‐TWA and PEL) for these substances. Therefore, we consider the risk from these substances to farmers handling pelleted seeds to be negligible.

## DISCLOSURE

*Approval of the research protocol*: The study protocol was approved by the Ethics Committee of Kochi University Medical School, Japan (approval number: 31‐171) on February 12, 2020. *Informed consent*: Informed consent for personal exposure measurement in the simulated workplace was acquired from experimenters representing farmers. Factory workers were offered the opportunity to opt‐out of the study. *Registry and the registration no*. *of the study* */* *trial*: This study is registered in the UMIN Clinical Trials Registry (https://www.umin.ac.jp/ctr/, UMIN000040090). *Animal studies*: N/A. *Conflict of interest*: This study was partly funded by the Japan Seed Trade Association. Author MT is a former member of the Japan Seed Trade Association.

## AUTHOR CONTRIBUTIONS

Masaichi Terada and Narufumi Suganuma conceived the idea; Masamitsu Eitoku, Naw Awn J‐P, Yukiko Iida, Masaichi Terada, and Narufumi Suganuma collected the data; Mitsugu Hirano, Masamitsu Eitoku, Yukiko Iida, and Masaichi Terada analyzed the data; Mitsugu Hirano, Etongola Papy Mbelambela, Masamitsu Eitoku, Naw Awn J‐P, Yukiko Iida, and Narufumi Suganuma contributed to the writing of the manuscript.

## Supporting information

Supplementary Material

## Data Availability

Research data are not shared.

## References

[1] Schenker M . Exposures and health effects from inorganic agricultural dusts. Environ Health Perspect. 2000;108(suppl 4):661‐664. 10931784 10.1289/ehp.00108s4661 PMC1637665

[2] Swanepoel AJ , Rees D , Renton K , Swanepoel C , Kromhout H , Gardiner K . Quartz exposure in agriculture: literature review and South African survey. Ann Occup Hyg. 2010;54(3):281‐292. 20172918 10.1093/annhyg/meq003

[3] Schenker MB , Christiani D , Cormier Y , et al. Respiratory health hazards in agriculture. Am J Respir Crit Care Med. 1998;158(5 II):S1‐S76. 10.1164/ajrccm.158.supplement_1.rccm1585s1 9817727

[4] Bignon J , Sebastien P , Gaudichet A , Jaurand MC . Biological effects of attapulgite. IARC Sci Publ. 1980;30:163‐181. 7239636

[5] Some silicates. IARC Monogr Eval Carcinog Risks Hum. 1997;68:243‐333. 9268739 PMC7681613

[6] Japanese Standards Association . JIS A 1481‐2: Determination of Asbestos in Building Material Products‐Part 2: Sampling and Qualitative Analysis for Judgement of Existence of Containing Asbestos (in Japanese). 2016. Accessed June 23, 2021. https://kikakurui.com/a1/A1481‐2‐2016‐01.html

[7] Japanese Standards Association . JIS K 3850‐1: Determination of Airborne Fibrous particles−Part 1: Optical Microscopy Method and Scanning Electron Microscopy Method (in Japanese). 2006. Accessed June 23, 2021. https://kikakurui.com/k3/K3850‐1‐2006‐01.html

[8] International Commission on Radiological Protection (ICRP) . Human Respiratory Tract Model for Radiological Protection, Publication 66. Oxford; 1994.

[9] Hoseki Y . Effects of the development of a machine system on the expansion of vegetable farming and management: using Hokkaido Onion farming as an ingredient (in Japanese). Jpn J Farm Manage. 2004;30:41‐58.

[10] Hokkaido vegetable map (in Japanese). Vol 40: JA Hokkaido Chuokai; 2017.

[11] Japan Association for Working Environment Measurement . The Primary Report on Results of Investigations Made on the Ideal Regulation of Airborne Toxic Substances at the Workplace; Survey on Specific Methods such as B Measurement (in Japanese). 1980.

[12] Ministry of Labour . Working Environment Measurement System in Japan (3rd Ed). Japan Association for Working Environment Measurement; 1996.

[13] Takahashi K , Higashi T . The development and regulation of occupational exposure limits in Japan. Regul Toxicol Pharmacol. 2006;46(2):120‐125. 16510222 10.1016/j.yrtph.2006.01.009

[14] Taylor AG , Eckenrode C , Straub R . Seed coating technologies and treatments for onion: challenges and progress. HortScience. 2001;36(2):199‐205.

[15] The Japan Society for Occupational Health . Guideline on personal exposure measurement of chemical substances (in Japanese). Sangyo Eiseigaku Zasshi. 2015;57:A13‐A60.

[16] Yamamoto S , Natsumeda S , Hara K , Yoshida S , Sakurai H , Ichiba M . Applicability of concentrations obtained by working environment measurement to assessment of personal exposure concentrations of chemicals. J Occup Health. 2014;56(2):85‐92. 24430837 10.1539/joh.12-0243-oa

[17] Nogami S , J‐p NA , Nogami M , et al. Radiographic diagnosis of pneumoconioses by AIR pneumo‐trained physicians: comparison with low‐dose thin‐slice computed tomography. J Occup Health. 2020;62(1):e12141. 33176059 10.1002/1348-9585.12141 PMC7384989

[18] International Labour Organization . Guidelines for the Use of the ILO International Classification of Radiographs of Pneumoconioses, Revised edition 2011. International Labour Organization.

[19] The Japan Society for Occupational Health . Recommendations of the occupational exposure limit for chemical substance (in Japanese). Sangyo Eiseigaku Zasshi. 2020;62(5):198‐230. 33041273 10.1539/sangyoeisei.S20001

[20] The Japan Society for Occupational Health . Reasons for the proposal of provisional values (2000) for assessment corresponding to excess lifetime risk levels of carcinogens (in Japanese). Sangyo Eiseigaku Zasshi. 2000;42(4):177‐192.

[21] Environment Agency . Future Measures against Hazardous Air Pollutants. [Kankyou to Sokutei Gijutsu]. (Second Report) (in Japanese). 1996;23(11):8–23.

[22] Q32: Q & A on chemical substance countermeasures (related to risk assessment) (in Japanese). https://www.mhlw.go.jp/stf/newpage_11389.html

[23] Mizuho Information & Research Institute Co., Ltd . Business Report for Development of Simplified Risk Assessment Support Tools (in Japanese). 2018;27‐31.

[24] Hashimoto H , Goto T , Nakachi N , et al. Evaluation of the control banding method–comparison with measurement‐based comprehensive risk assessment. J Occup Health. 2007;49(6):482‐492. 18075208 10.1539/joh.49.482

[25] Occupational Safety and Health Administration . Assigned Protection Factors for the Revised Respiratory Protection Standard. 2009. Accessed June 11, 2021. https://www.mhlw.go.jp/content/11201000/000576491.pdf

[26] Japanese Standards Association . JIS T 8150: Guidance for selection, use and maintenance of respiratory protective devices (in Japanese). 2006. Accessed June 11, 2021. https://kikakurui.com/t8/T8150‐2006‐01.html

[27] Barnes H , Goh NSL , Leong TL , Hoy R . Silica‐associated lung disease: an old‐world exposure in modern industries. Respirology. 2019;24(12):1165‐1175. 31517432 10.1111/resp.13695

[28] Smit L , Wouters I , Hobo M , Eduard W , Doekes G , Heederik D . Agricultural seed dust as a potential cause of organic dust toxic syndrome. Occup Environ Med. 2006;63(1):59‐67. 16361407 10.1136/oem.2005.021527 PMC2078022

