# Supplementary material for: Risk assessment of farmers handling pelleted seeds containing crystalline silica and attapulgite
Source: J Occup Health. 2021 Dec 10;63(1):e12304. doi: 10.1002/1348-9585.12304 (PMC8662661; doi:10.1002/1348-9585.12304)
Supplement: Supplementary file 1 — Supplementary Material [file JOH2-63-e12304-s001.docx]

a b


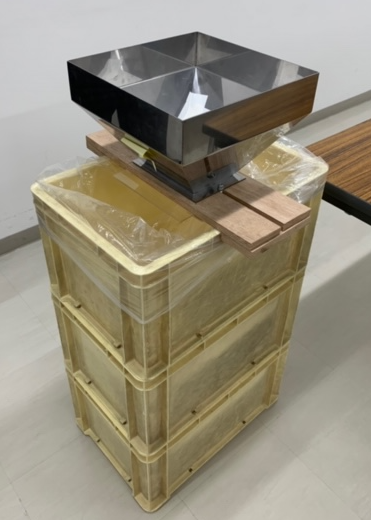


c d

**Supplementary Figure 1** Exposure assessment in a simulated workplace.

(a) A pseudo-device simulating (b) a standard seeding machine (High Speed Onion Spreader OSE-110). The hopper of the pseudo-device was installed at the same height as that of the standard machine. (c) A high-volume sampler (HVS) was installed at a height of 1,200 mm and at a distance of 300 mm from the dust source. The HVS sampled 30 m^3^ of air in 1 h. (d) Experimenter representing a farmer wore two personal samplers, one for attapulgite and one for dust, that sampled 0.48 and 1.2 m^3^ of air in 8 h, respectively. All dimensions in (c) and (d) are shown in millimeters.

**Supplementary Table 1.** Summary of methods used for assessment of exposure to attapulgite, total dust, respirable dust, and respirable crystalline silica

| Target | Method | Detection limit | Apparatus |
| --- | --- | --- | --- |
| ***Analysis of bulk pelleted seeds*** |  |  |  |
| Attapulgite | Dispersion staining-phase contrast microscopy: JIS A 1481-2 (2016) | -  (Qualitative analysis) | BX-51 microscope (Olympus Corporation, Tokyo, Japan) |
|  | X-ray diffraction | -  (Qualitative analysis) | X'pert^3^ Powder diffractometer (PANalytical, Almelo, The Netherlands) |
| Silica content (%) | X-ray diffraction | 0.0228 (mg) | X'pert PRO MPD diffractometer (PANalytical) |
| ***Airborne concentration measurement in the simulated workplace*** | | | |
| Sampling |  |  |  |
| Respirable dust | High-volume air sampling  Particle size separation: PM4 (4 μm, 50% cutoff) | - | HV-500F air sampler (Sibata Scientific Technology Co., Ltd., Saitame, Japan)  HV-500PM4 granulation device (Sibata Scientific Technology Co.) |
| Analysis |  |  |  |
| Respirable dust (mg/m^3^) | Gravimetry | 0.01 (mg)^†^ | HM-202 balance (A&D Co., Ltd., Tokyo, Japan) |
| Silica content (%) | X-ray diffraction | 0.0228 (mg) | X'pert PRO MPD diffractometer (PANalytical) |
| ***Personal exposure measurement in the simulated workplace*** | | | |
| Sampling |  | - |  |
| Attapulgite | Air sampling  Filtration collection (pore size 0.8 μm) | - | MP-W5P sampler (Sibata Scientific Technology Co., Ltd.)  225-321 filter holder with a membrane filter (SKC Ltd., Blandford Forum, UK) |
| Total dust & respirable dust | Air sampling  Inertial collision particle size selection (4 μm, 50% cut)  Filtration collection of respirable dust | - | MP-W5P sampler (Sibata Scientific Technology Co., Ltd.)  NWPS-254 filter holder (Sibata Scientific Technology Co., Ltd.)  TF98R filter (Sibata Scientific Technology Co., Ltd.) |
| Analysis |  |  |  |
| Attapulgite (fibers/L) | Dispersion staining-phase contrast microscopy: JIS K 3850-1 (2006) | 0.5 (fibers/L)^‡^ | BX-51 microscope (Olympus Corporation) |
| Total dust (mg/m^3^) | Gravimetry | 0.001 (mg)^§^ | MC-5 balance (Sartorius AG, Goettingen, Germany) |
| Respirable dust (mg/m^3^) | Gravimetry | 0.001 (mg)^§^ | MC-5 balance (Sartorius AG) |

^†^ Sampling volume 30 m^3^.

^‡^ Sampling volume 0.48 m^3^; calculation formula 8 h × 60 min/h × 1 L/min.

^§^ Sampling volume 1.2 m^3^; calculation formula 8 h × 60 min/h × 2.5 L/min.
